# Déterminants de la non-observance au traitement antirétroviral chez l’adulte à Kinshasa

**DOI:** 10.11604/pamj.2020.37.157.13261

**Published:** 2020-10-14

**Authors:** Benilde Bepouka Izizag, Hippolyte Situakibanza, Florian Kiazayawoko, Aliocha Nkodila, Eric Mafuta, Philippe Lukanu, Henry Mukumbi, Murielle Longokolo, Madone Mandina, Nadine Mayasi, Amede Kinuka, Evelyne Amaela, Willy Kazadi, Marcel Mbula

**Affiliations:** 1 Service des Maladies Infectieuses et Tropicales, Département de Médecine Interne, Cliniques Universitaires, Faculté de Médecine, Université de Kinshasa, Kinshasa, République Démocratique du Congo,; 2 Cités des Aveugles, Kinshasa, République Démocratique du Congo,; 3 Ecole de Santé Publique, Faculté de Médecine, Université de Kinshasa, Kinshasa, République Démocratique du Congo,; 4 Département de Médecine de Famille, Université Protestante au Congo, Kinshasa, République Démocratique du Congo,; 5 ACS AMO-CONGO (ONG-ASBL/Santé), Kinshasa, République Démocratique du Congo,; 6 Cliniques Rapha, Kinshasa, République Démocratique du Congo

**Keywords:** Traitement antirétroviral, non-observance, déterminants, Kinshasa, Antiretroviral therapy, non-compliance, determinants, Kinshasa

## Abstract

**Introduction:**

l´objectif de cette étude était d´identifier les déterminants de non-observance des patients vivant avec le VIH (PVVIH) au TAR (traitement antirétroviral) à Kinshasa.

**Méthodes:**

dans une étude transversale conduite à Kinshasa du 1^e^ ^r^mai au 31 août 2015 chez des PVVIH âgées d´au moins 18 ans et sous traitement antirétroviral depuis au moins 3 mois. Un échantillon probabiliste de 400 patients a été pris en compte. Le CASE Adherence Index (méthode subjective) et le renouvellement d´ordonnance (méthode objective) ont évalué l´observance. Les déterminants de la non-observance ont été recherchés par régression logistique multiple.

**Résultats:**

les 400 PVVIH avaient un âge médian de 43 ans (18-75). La fréquence de non-observance globale était de 25,5%. La fréquence de la non-observance objective était plus élevée que celle de la non-observance subjective (29% vs 21%, p = 0,01). Le paiement de la consultation (ORaj: 1,70; IC95%: 1,02-2,81; p = 0,042), les effets indésirables (ORaj: 2,23; IC95%: 1,33-3,75; p = 0,002) et le manque de perception tel que l´oubli d´une dose qui peut aggraver la maladie (ORaj: 4,16; IC95%: 1,04-16,68; p=0,045) ont émergé comme déterminants de la non-observance. La présence d´une personne de confiance était un facteur protecteur contre la non-observance (ORaj: 0,54; IC95%: 0,39-0,93; p = 0,004).

**Conclusion:**

la fréquence de la non-observance au TAR demeure élevée à Kinshasa. La différence de fréquence entre l´appréciation objective et subjective de l´observance indique l´importance de la biologie dans le suivi des PVVIH sous antirétroviraux. La prise en compte des déterminants sera nécessaire pour définir des stratégies qui permettront l´amélioration de l´observance.

## Abstract (en)

**Introduction:**

the purpose of this study was to identify the determinants of non-compliance of persons living with HIV with antiretroviral treatment in Kinshasa.

**Methods:**

we conducted a cross-sectional study in Kinshasa from 1^st^ May to 31^st^ August 2015. The study population was composed of patients aged at least 18 years living with HIV who had been treated with antiretroviral drugs for at least 3 months. Adherence Index (subjective method) and prescription refills (objective method) were used to assess compliance. Determinants of non-compliance were identified by logistic regression model.

**Results:**

the 400 patients living with HIV had a median age of 43 years (18-75). Global non-compliance rate was 25.5%. Objective non-compliance rate was higher than that of subjective non-compliance (29% vs 21%, p = 0.01). Payment for consultation [adjusted odds ratio (AOR): 1.70; 95% confidence interval (95% CI): 1.02-2.81; p = 0.042), adverse reactions (AOR: 2.23; 95% CI: 1.33-3.75; p = 0.002) and the lack of awareness that missing a dose may worsen disease (AOR: 4.16; 95% CI: 1.04-16.68; p = 0.045) were determinants of non-compliance. Having trusted person was a protective factor versus non-compliance (AOR: 0.54; 95% CI: 0.39-0.93; p = 0.004).

**Conclusion:**

the rate of non-compliance with antiretroviral treatment is high in Kinshasa. The evaluation of determinants is necessary to establish strategies for improving compliance.

## Introduction

Le monde était sur le point de fournir une thérapie antirétrovirale à 15 millions de personnes en fin 2015. En 2012, 9,7 millions de personnes ont suivi cette thérapie dans les pays à revenu faible et intermédiaire, soit 61% des personnes éligibles selon les directives de 2010 de l´Organisation Mondiale de la Santé (OMS). Entre 1996 et 2012, la thérapie antirétrovirale a permis d´éviter 6,6 millions de décès liés au sida dans le monde, dont 5,5 millions dans les pays à revenu faible et intermédiaire [1]. L´avènement des médicaments antirétroviraux pour le traitement de l´infection à virus de l´immunodéficience humaine (VIH) est l´un progrès les plus remarquables de la médecine. Ces molécules ont prouvé leur efficacité dans la réduction de la charge virale et l´amélioration clinique [2]. Le traitement antirétroviral (TAR) entraine la diminution de la réplication virale, de la mortalité et la restauration de l´immunité [3].

Parmi les patients vivant avec le VIH (PVVIH), seul environ un tiers prend ses médicaments selon la prescription. Cependant même lorsque les patients comprennent pleinement les conséquences de la non-observance au traitement, les taux d´observance ne sont pas optimaux. La bonne observance est un facteur décisif dans le succès du traitement. Contrairement à d´autres maladies chroniques, la vitesse de réplication et la mutation rapide du VIH exigent des niveaux très élevés d´observance (soit = 95%) pour obtenir la suppression durable de la charge virale. La mauvaise observance peut rapidement conduire à la résistance et le virus peut ensuite être transmis à d´autres personnes [4].

De nombreuses études expérimentales et d´observation effectuées essentiellement dans les pays développés ont montré l´importance de l´observance comme facteur majeur de l´efficacité thérapeutique. Mills *et al*. en 2006, dans une méta-analyse, ont trouvé un combiné continental (Afrique et Amérique du nord) de non-observance au traitement antirétroviral de 36%, avec 45% de non-observance en Amérique du Nord et 23% en Afrique [5]. Vingt-deux pour cent de non-observance ont été rapportés en Côte d´Ivoire [6], 13% au Cameroun [7].

Byakika *et al*. [8] ont rapporté 32% de non-observance au TAR en Ouganda. Quarante-six pour cent de non-observance au Nigéria a été rapporté par Iliyasu *et al*. [9] et 37% en Afrique du Sud par Orell *et al*. [10]. En République Démocratique du Congo (RDC), l´épidémie de VIH est relativement stable, avec une prévalence de la maladie variant autour de 1,2% en 2013 [11] dont seuls 12,3% des patients éligibles ont eu accès aux traitements antirétroviraux en 2012 [12]. Une étude menée à Kinshasa en 2012 a rapporté un taux de non-observance de 20,9% [13].

La non-observance au traitement antirétroviral a souvent été associée à divers facteurs dont ceux liés aux p

atients, au système des soins de santé, au traitement et aux facteurs socio-économiques [5-10, 13].

Les objectifs spécifiques de cette étude étaient de: décrire les caractéristiques sociodémographiques des PVVIH sous antirétroviral (ARV) dans la ville de Kinshasa; déterminer la fréquence des patients non-observants au traitement antirétroviral; identifier les facteurs associés à la non-observance au traitement antirétroviral.

## Méthodes

Il s´agit d´une étude transversale à visée analytique qui s´est déroulée du 1^er^ mai au 31 aout 2015. Elle a été menée auprès des personnes vivant avec le VIH dans 9 structures sanitaires tirées au sort sur une liste des 63 structures ayant une file active d´au moins 100 patients sous ARV sur les 365 structures de prise en charge à Kinshasa. Les critères d´inclusion étaient: 1) être âgé d´au moins 18 ans et sous ARV depuis au moins 3 mois; 2) avoir donné son consentement éclairé. Les critères d´exclusion étaient: 1) avoir un trouble de comportement; 2) être hospitalisé.

**Taille de l´échantillon:** la taille de l´échantillon est estimée à partir de la formule suivante: n = (Z^2 pq)/d^2, avec p: la proportion des PVVIH considérées comme non-observantes; 20,9% à Kinshasa [13], q: la proportion des PVVIH considérées comme observantes, a: le risque d´erreur (0,05), d: le degré de précision (0,05), Z: le coefficient de confiance pour un degré de confiance de 95% (1,96). La taille est calculée pour un intervalle de confiance de 95% et un degré de précision de 0,05. En tenant compte du refus et des défauts d´enregistrement, cette taille minimale sera majorée d´au moins de 10%, soit 280.

Un total de 422 PVVIH ont été sélectionnées et ont été réparties proportionnellement en fonction de la file active de chaque structure. La collecte des données de cette étude a utilisé deux techniques, soient des entrevues face-à-face et une revue documentaire. Le questionnaire était constitué de trois types de questions: ouverte, fermée et semi-fermée. Il était systématiquement proposé à chaque patient venu renouveler son ordonnance ou pour une visite médicale de routine, de participer à l´enquête. Les variables enquêtées comprenaient les caractéristiques sociodémographique, les facteurs socio-culturels, la perception du VIH et des ARV, ainsi que les facteurs liés au traitement. Les entretiens ont été conduits en français et/ou en lingala par des enquêteurs préalablement formés. L´analyse des registres de la pharmacie a permis de recueillir pour la population d´étude, les informations relatives à la date de début du traitement et à la fréquence d´approvisionnement en ARV. Celle des dossiers médicaux a permis de confirmer les informations sociodémographiques et les informations sur le traitement.

Pour mesurer l´observance, nous avons utilisé deux méthodes.

### Méthode subjective

Le CASE Adherence Index: c´est une méthode de mesure d´observance utilisant un questionnaire développé par le New York Academy of Medicine (NYAM) [14]. Il se compose de trois questions à savoir Q1: avez-vous la difficulté à prendre votre médicament à l´heure? 4 points: jamais, 3 points: rarement, 2 points: la plupart, 1 point: tout le temps; Q2: combien de jours par semaine diriez-vous que vous avez manqué au moins une dose de vos médicaments ARV? 1 point: tous les jours, 2 points: 4 à 6 jours par semaine, 3 points: 2 à 3 jours par semaine, 4 points: une fois par semaine, 5 points: moins d´une fois par semaine, 6 points: jamais; Q3: à quand remonte la dernière fois que vous avez raté au moins une dose de médicament contre le VIH? 1 point: la semaine passée, 2 points: il y a 1-2 semaine, 3 points: il y a 3-4semaines, 4 points: entre 1-3 mois passé ,5 points: plus de 3 mois, 6 points: jamais. Les scores des patients dans le CASE Adhérence Index étaient additionnés pour obtenir un score composite qui variera de 3 à 16 points. Les patients dont l´index était = 10 points étaient classés non-observants et ceux avec un score > 10 observants.

### Méthode objective

Observance calculée: mesurée par le rapport entre le nombre d´ordonnances dispensées et la quantité théorique d´ordonnances attendues (lequel correspond au nombre de mois de suivi du traitement). Le patient était considéré comme non observant lorsque ce rapport était inférieur à 0,95 [15, 16].

### Observance globale

Mesurée par l´addition des résultats de l´observance subjective et objective (considérant 0 = non observant et 1 = observant). Si l´addition est = 1, le patient est considéré comme non-observant et si l´addition donne 2, le patient est considéré comme observant [17].

### Traitement et analyse des données

Les données ont été saisies sur Excel 2010 et l´analyse a été effectuée en utilisant le logiciel SPSS version 21. Les analyses descriptives effectuées ont inclus la mesure des proportions pour les variables discrètes, la moyenne, écart type pour les données quantitatives à distribution Gaussienne et la médiane (EIQ = écart type interquartile) pour les données quantitatives à distribution non-Gaussienne. Le test Khi-2 de Pearson a été effectué pour évaluer la dépendance des variables qualitatives à la non-observance. Le test t de Student a été effectué pour comparer les moyennes pour les données normalement distribuées. La régression logistique multiple a été utilisée pour rechercher les déterminants de la non-observance globale. Ceux qui avait une valeur significative en univarié sont retenu en multivarié.

## Résultats

### Caractéristiques sociodémographiques de la population d´étude

Parmi les 422 patients éligibles à l´enquête, 400 ont été interrogés, soit une exhaustivité globale de 95%. Ce collectif comptait 309 femmes et 91 hommes, d´âge médian de 43 ans avec des extrêmes de 18 et 75 ans. Les patients étaient majoritairement mariés (41%). La plupart (65,4%) avait un niveau d´étude secondaire. La moitié des patients (50%) priait à l´église de réveil. Les détails des caractéristiques de la population générale sont résumés dans le Tableau 1.

**Tableau 1:** caractéristiques sociodémographiques de la population d’étude

Variable	Tous N=400 (%)
**Sexe**	
Masculin	91(22,8)
Féminin	309(77,3)
**Age, ans**	
xET	45,04-10,6
<30	24(6,0)
30-39	100(25,0)
40-49	133(33,3)
50-59	103(25,8)
>60	40(10,0)
**Etat civil**	
Célibataire	85(21,3)
Marié	164(41,0)
Divorcé	56(14,0)
Veuf	95(23,8)
**Niveau instruction**	
Sans instruction	22(5,5)
Primaire	50(12,5)
Secondaire	261(65,4)
Supérieur	66(16,5)
**Religion**	
Catholique	77(19,3)
Protestante	57(14,3)
Kimbaguiste	14(3,5)
musulmane	4(1,0)
Eglise de réveil	200(50,0)
Autres	48(12,0)

### Fréquence de non-observance

Dans cette étude, 3 catégories d´observance ont été considérées: subjective, objective et globale. Sur 400 PVVIH suivies durant la période d´étude, 85, 117 et 102 avaient respectivement présenté une non-observance subjective, objective et globale au traitement aux ARV. En outre, La différence entre la non-observance subjective (21,2%) et objective (28%) était statistiquement significative (p = 0,01).

### Raisons de la non-observance

**Figure 1:**
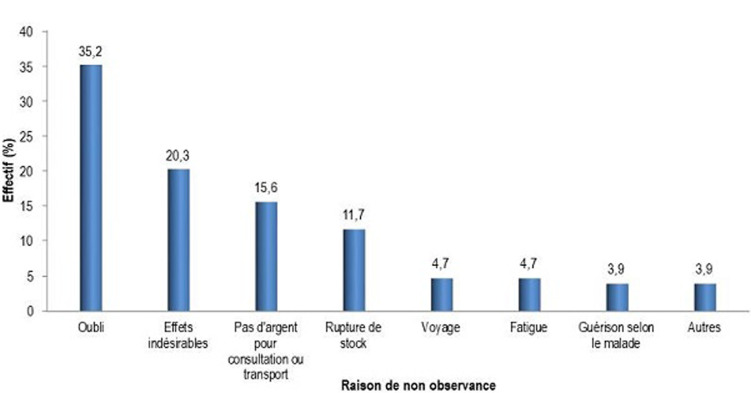
raison de non-observance

La Figure 1 présente les différentes raisons de non-observance. Elle montre que l´oubli était la raison de non-observance la plus évoquée chez les patients (soit chez 45 PVVIH).

### Facteurs associés à la non-observance en analyse univariée

En analyse univariée, le jeune âge (p = 0,027), prise de tabac (p = 0,037), la longue durée du traitement (p = 0,011), la longue durée de la maladie (p = 0,014), la présence des effets indésirables (0,002), l´interruption de traitement (p = 0,001), la faible amélioration de l´état depuis le début du traitement (p = 0,003), le manque d´information du traitement par des personnes de confiance (p = 0,012) et la perception qu´oublier une dose n´est pas nocive (p = 0,003) sont des facteurs associés à la non-observance (Tableau 2).

**Tableau 2:** répartition des sujets en fonction des facteurs associés ou non à la non-observance en analyse univariée

Variable	Tous N=400 (%)	Non-observant n=102 (%)	Observant n=298 (%)	p
Revenu mensuel				0,196
<60 USD	203(50,8)	56(54,9)	147(49,3)	
≥60 USD	197(49,3)	46(45,1)	151(50,7)	
Payement de consultation				0,143
Non	224(56,0)	52(51,0)	172(57,7)	
Oui	176(44,0)	50(49,0)	126(42,3)	
Habite avec				
Famille	351(87,8)	90(88,2)	261(87,6)	0,984
Ami	12(3,0)	3(2,9)	9(3,0)	
Seul	37(9,3)	9(8,8)	28(9,4)	
Statut VIH divulgué par la communauté	47(11,8)	14(13,7)	33(11,1)	0,289
Statut VIH divulgué par la famille	196(49,0)	53(52,0)	143(48,0)	0,282
Rappel de la dose				0,199
Soi	347(86,8)	91(89,2)	256(85,9)	
Famille	39(9,8)	6(5,9)	33(11,1)	
Ami	14(3,5)	5(4,9)	9(3,0)	
Soutient social				0,347
Oui	193(48,3)	47(46,1)	146(49,0)	
Non	207(51,8)	55(53,9)	152(51,0)	
Prise d’alcool	45(11,3)	14(13,7)	31(10,4)	0,228
Prise de tabac	32(8,0)	13(12,7)	19(6,4)	**0,037**
Insécurité alimentaire	225(56,3)	64(62,7)	161(54,0)	0,078
Durée du traitement (mois)	59,7?39,1	65,0?39,6	57,9?38,9	**0,011**
Durée de la maladie	65,0?40,7	70,1?42,1	63,1?40,1	**0,014**
Effets indésirables	106(26,5)	39(38,2)	67(22,5)	**0,002**
Suivre un autre traitement	88(22,0)	18(17,6)	70(23,5)	0,137
Interruption de TAR	128(32,0)	4645,1)	82(27,5)	**0,001**
Connaissance du risque de ne pas suivre le traitement	353(88,3)	86(84,3)	267(89,6)	0,107
Faible amélioration	46(11,6)	18(17,6)	28(9,3)	**0,003**
Absence d’info sur traitement	54(13,5)	19(18,6)	35(11,7)	0,198
Connaissance du traitement par le conjoint	125(79,6)	35(83,3)	90(78,3)	0,324
Information du traitement par des personnes de confiance	267(66,8)	78(76,5)	189(63,4)	**0,012**
Courte interruption n’est pas nocive	51(12,8)	14(13,7)	37(12,4)	0,821
Oublier une dose n’est pas nocif	65(16,3)	26(25,5)	39(13,1)	**0,003**
Oublier une dose peut aggraver la maladie	315(78,8)	75(73,5)	24(80,5)	0,207
ARV doit être pris à vie	352(88,0)	84(82,4)	268(89,9)	0,052

### Déterminants de la non-observance

En analyse multivariée, la force d´association observée en analyse univariée n´a persistée que pour le paiement de la consultation, l´absence de connaissance qu´oublier une dose peut aggraver la maladie, l´information du traitement à des personnes de confiance et les effets indésirables. Ces facteurs ont donc émergé comme les principaux déterminants de la non-observance du TAR. Le risque était multiplié par 2 chez les sujets qui payaient les consultations (OR ajusté 1,703 IC à 95% [1,020- 2,843], p = 0,042) et qui avaient des effets indésirables (OR ajusté 2,230 IC à 95% [1,327-3,747], p = 0,002), par 4 chez les sujets n´ayant pas de connaissance qu´oublier la dose peut aggraver la maladie (OR ajusté 4,156 IC à 95% [1,035-16,678], p = 0,045). En revanche, le coefficient β étant négatif, le risque de non-observance est réduit de 2 chez les sujets dont les personnes de confiance sont informés du traitement (OR ajusté 0,539 IC à 95% [0,929-2,548], p = 0,004) (Tableau 3).

**Tableau 3:** déterminants de la non-observance globale en analyse multivariée

Variables	β	p	ORaj	IC 95%	
Amélioration de l’état depuis le début du traitement					
Oui			1		
Non	0,620	0,398	1,859	0,441	7,834
Il faut payer la consultation					
Non			1		
Oui	0,532	**0,042**	1,703	1,020	2,843
ARV doit être pris à vie					
Oui			1		
Non	0,622	0,065	1,863	0,961	3,611
Interrompre le TAR					
Non			1		
Oui	0,324	0,199	1,383	0,844	2,267
Oublier une dose peut aggraver la maladie					
Oui			1		
Non	1,424	**0,045**	4,156	1,035	16,678
Personne de confiance informe de du traitement					
Oui			1		
Non	-0,431	**0,004**	0,539	0,929	2,548
Effets indésirable					
Non			1		
Oui	0,802	**0,002**	2,230	1,327	3,747
Absence d’information par le médecin					
Oui			1		
Non	0,376	0,140	1,457	,883	2,402
Constante	-5,174	**0,000**	0,006		

β= coefficient de régression logistique. OR = Odds ratio. IC95% = Intervalle de confiance

## Discussion

Le présent travail a cerné les déterminants de non-observance au traitement antirétroviral chez l´adulte à Kinshasa. Les objectifs spécifiques retenus étaient de décrire les caractéristiques des PVVIH sous ARV, déterminer la fréquence des non-observants et d´identifier les facteurs associés à la non-observance au traitement antirétroviral. Le niveau de non-observance objective était plus élevé que le niveau de non-observance subjective (29% vs 21%, p = 0,01).Cela serait dû au fait que beaucoup de patients surestiment leur observance par la déclaration mais après vérification, nous constatons que la fréquence de renouvellement d´ordonnance n´est pas optimale. Le même constat a été fait par Musumari *et al*. Issifou *et al*. Mbopi *et al*. Essomba *et al*. Kanté *et al*. et Folefack *et al*. [13, 15-19]. Nous avons un taux de non-observance globale de 25,5%. Ces résultats sont corroborés par les résultats de la prévalence continentale (23%) de l´Afrique et les travaux de Musumari *et al*. Saha *et al*. Oku *et al*. et Wakibi *et al*. [13, 20-22]. La différence avec Issifou *et al*. Silva *et al*. et Bayew *et al*. serait due à une différence méthodologique [17, 23, 24].

Les raisons de non-observance de la présente étude étaient: oubli, effets indésirables, manque d´argent pour payer le transport ou la consultation, rupture de stock, voyage, fatigue, guérison selon les patients, voyage; Musumari *et al*. a trouvé comme raisons: oubli, difficulté à payer le transport et la consultation, rupture de stock, défaut de nourriture, voyage, fatigue, alcool, loin de la maison, effet secondaire, fatigué, déprimé [13]; Wakibi *et al*. a trouvé comme raisons: oubli, RDV manqué, rupture de stock, dépression, colère, ES, mauvaise attitude envers ARV [22]. Les raisons évoquées dans l´étude d´Amberbir *et al*. étaient les suivantes: oubli, maladie, occupation, stigmatisation [25].

L´oubli était la cause la plus évoquée de non-observance. Ces résultats diffèrent de ceux observés dans les pays du Nord où les motifs liés aux médicaments, ont été rapportés comme premières causes de non-observance [26, 27]. L´engagement du patient lui-même à suivre son traitement semble être le principal déterminant de l´observance, suivi des facteurs institutionnels comme l´illustre l´effet des ruptures de stocks en médicaments. Le rôle prédictif de la composante socio-économique sur la non-observance est minoré dans cette cohorte, sans doute á cause de la politique nationale de la gratuité des ARV visant à assurer l´équité d´accès pour tous les nécessiteux au TAR. Dans ce cas, le conseil de l´observance pourrait intégrer des stratégies, comme l´utilisation des aides de mémoire, pour éviter d´oublier la prise des pilules.

La non-observance était associée au jeune âge. Nos résultats se rapprochent aux résultats de Bayew *et al*. et Linda *et al*. cohorte française APROCO [24, 28, 29]. Cela serait dû au manque d´expérience de la maladie chez les personnes jeunes. La non-observance était associée à la prise de tabac. Ce résultat est corroboré par les résultats de Oku *et al*. Degroote *et al*. et Shigdel *et al*. [21, 30, 31]. D´autre part, les risques de non-observance chez les patients qui prenaient le tabac sont en accord avec la littérature [32, 33]. Les patients qui prenaient le tabac sont nettement moins engagés à leur fournisseur de soins de santé, et sont susceptibles de présenter un refus de traitement [34, 35].

La longue durée de traitement était associée à la non-observance. Ce résultat est corroboré par le résultat de Mbopi *et al*. [15]. La durée de traitement est décrite comme un facteur de lassitude dans plusieurs études dont l´étude d´Andréo *et al*. [36]. La longue durée de la maladie était associée à la non-observance. Ce résultat rejoint celui de Silva *et al*. Cela est dû au fait que plus la maladie est chronique moins est le respect des recommandations du médecin [23].

Les patients qui avaient les effets secondaires étaient moins observants que ceux qui n´en avaient pas. Ces résultats se rapprochent des résultats de Issifou *et al*. Saha *et al*. et Silva *et al*. [17, 20, 23]. Dans plusieurs recherches, les auteurs s´accordent pour dire que plus les effets indésirables sont importants, moins le patient en aura plus il aura tendance à être observant [36, 37] Les patients qui avaient une faible amélioration de l´état depuis le début des ARV étaient moins observants. Cette étude était en accord avec une autre étude [21] où rester en bonne santé était un facteur clé de motivation de l´observance du traitement mais, Olowookere *et al*. dans le Sud-Ouest du Nigeria a rapporté que se sentir en bonne santé, était un facteur de risque de non-observance. Il a en outre indiqué que la plupart des patients ont tendance à abandonner le traitement une fois qu´il y avait une amélioration de leur état de santé [38].

Les patients qui n´informaient pas aux personnes de confiance de leur maladie étaient moins observants que ceux qui les informaient. Ce résultat est corroboré avec le résultat de Folefack *et al*. [19]. Les patients qui avaient la perception qu´oublier une dose n´est pas nocive étaient moins observants que ceux qui percevaient qu´oublier une dose est nocive. Ce résultat rapproche les résultats de Musumari *et al*. et Wakibi *et al*. [13, 22]. Un certain nombre d´études trouvent un lien entre perception de la sévérité de la maladie et prise des traitements. Les patients qui ont une bonne connaissance de leur maladie ont tendance à accepter la maladie, ses effets et son traitement et ont davantage de chance d´être observants. Le niveau de la croyance dans les avantages et l´efficacité des ARV pourrait également promouvoir l´observance, comme documenté par plusieurs études [39, 40].

Il convient de signaler que lors de l´analyse multivariée par régression logistique, trois facteurs ont émergés comme déterminants de la non-observance au traitement antirétroviral. Il s´agit du paiement de la consultation, des effets indésirables et du manque de perception qu´oublier une dose peut aggraver la maladie, avec un risque multiplié respectivement par 2 et 4 tandis que l´information du traitement par une personne de confiance est protecteur contre la non-observance, avec un risque de non-observance réduit de moitié. Le paiement de la consultation a été aussi reconnu comme déterminant au Nigeria par Oku *et al*. [21]. La population congolaise sous ARV dans cette série ayant un faible revenu et des contraintes financières, a des difficultés à payer la consultation et ceci décourage les malades à être réguliers aux consultations, à l´approvisionnement des médicaments et à suivre avec régularité le traitement.

Les contraintes financières parmi les patients étaient un obstacle majeur à l´observance comme observé dans cette étude. Bien que les médicaments ont été donnés gratuitement dans la plupart de structure de prise en charge. Les effets indésirables sont aussi reconnus comme déterminants dans les études d´Issifou *et al*. Saha *et al*. et Silva *et al*. [17, 20, 23]. Les effets indésirables indisposent les patients et les dépriment, les empêchant de prendre régulièrement leurs ARV. Ceci rejoint les conclusions de plusieurs autres études [41-43]. En outre, une méta-analyse a montré que variant de légère à sévère et urgence à la chronicité, les effets indésirables des médicaments antirétroviraux étaient une raison importante de la non-observance [32]. La gestion des symptômes est un élément essentiel de la réussite de l´observance au TAR. Par conséquent, il est nécessaire de discuter de tous les effets indésirables potentiels avec les patients avant qu´ils ne commencent le TAR. Le manque de perception qu´oublier une dose peut aggraver la maladie avait deux fois plus de risque de non observance que ceux qui avaient cette perception. Ce même constat a été remarqué par Musumari *et al*. [13]. Le manque de connaissance sur la réplication virale et l´échec thérapeutique peut faire que le patient ne comprenne pas le risque d´oublier une dose. La connaissance du traitement par une personne de confiance était protectrice contre le non observance avec un risque de non-observance réduit de moitié. Ce même constat a été fait par Kanté [18]. La personne de confiance renforce l´observance en fortifiant le patient sur la prise des ARV.

### Force de l´étude

Cette étude tire ses données à partir d´un échantillon de participants choisis parmi les PVVIH de manière aléatoire dans 9 structures de prise en charge à Kinshasa. Les résultats de cette étude peuvent donc dans une large mesure représenter la situation des patients sous ARV à Kinshasa; la première étude en RDC à avoir utilisé la mesure d´observance appelée CASE Adherence Index; en identifiant les déterminants de non-observance, cette étude a rendu disponible une base de données utile pour améliorer la prise en charge des PVVIH à Kinshasa.

### Limite de l´étude

Cette étude a des limitations. Premièrement, le caractère transversal de l´étude masque le dynamisme de l´observance dans le temps. Deuxièmement, Les méthodes biologiques telles que la mesure de la charge virale ou le dosage des anti-protéases auraient garanti la validité de notre questionnaire.

## Conclusion

A la lumière de ces résultats, les conclusions ci-après peuvent être tirées: la prévalence de non-observance au TAR est encore élevée à Kinshasa. L´étude a trouvé une discordance entre les deux méthodes d´observance utilisées, ce qui souligne l´importance des méthodes biologiques. Le payement de consultation, les effets secondaires, le manque de perception qu´oublier une dose peut aggraver la maladie et la connaissance du traitement par une personne de confiance ont été identifiée comme déterminants de non-observance au TAR à Kinshasa.

### Etat des connaissances sur le sujet

- La fréquence de non-observance à Kinshasa est élevée;
- L´insécurité alimentaire est le principal facteur connu associé à la non-observance à Kinshasa;
- Il n´existe pas de méthode de référence pour mesurer l´observance, la littérature recommande de recourir à au moins deux méthodes, dont l´une doit reposer sur les déclarations du patient.

### Contribution de notre étude à la connaissance

- La première étude ayant utilisé comme méthode de mesure d´observance le CASE Adherence Index associé au renouvellement d´ordonnance;
- La personne de confiance est reconnue comme facteur protecteur face à la non-observance au TAR à Kinshasa;
- Le payement de consultation, les effets secondaires, le manque de perception qu´oublier une dose peut aggraver la maladie ont été identifié comme déterminants de non-observance au TAR à Kinshasa.
